# Use of perennial plants in the fight against gastrointestinal nematodes of sheep

**DOI:** 10.3389/fpara.2023.1186149

**Published:** 2023-06-02

**Authors:** Antonio Bosco, Antonello Prigioniero, Alessandra Falzarano, Maria Paola Maurelli, Laura Rinaldi, Giuseppe Cringoli, Giovanni Quaranta, Salvatore Claps, Rosaria Sciarrillo, Carmine Guarino, Pierpaolo Scarano

**Affiliations:** ^1^ Department of Veterinary Medicine and Animal Production, CReMoPAR - Centro Regionale per il Monitoraggio delle Parassitosi, University of Naples Federico II, Naples, Italy; ^2^ Department of Science and Technology, University of Sannio, Benevento, Italy; ^3^ Department of Mathematics, Computer Science and Economics, University of Basilicata, Potenza, PZ, Italy; ^4^ MEDES Foundation - Mediterranean Sustainable Development Foundation, Sicignano degli Alburni, SA, Italy; ^5^ CREA-ZA – Consiglio per la Ricerca e la Sperimentazione in Agricoltura, Centro di Ricerca Zootecnia e Acquacoltura, Bella Muro, PZ, Italy

**Keywords:** gastrointestinal nematodes (GIN), anthelmintic resistance, phytotherapy (edible plants), polyphenols, *Cichorium intybus* L., *Foeniculum vulgare* Mill., *in vitro* test

## Abstract

**Background:**

Gastrointestinal nematodes (GINs) are a serious problem in ruminant pastures worldwide. They generate production losses, from the point of view of both the food chain and animal mortality. This study provides preliminary results concerning the use of pasture plants in the Campania region (of southern Italy) to control GINs in sheep.

**Methods:**

Sixteen species of wild and cultivated perennial plants present in seminatural pastures were sampled. All species were extracted with the conventional maceration technique, using three different solvents (i.e., H_2_O, EtOH, and an EtOH:H_2_O (8:2) mixture) in order to extract different bioactive compounds. The total phenolic content (TPC; assessed *via* the Folin–Ciocȃlteu assay) of all extract samples was preliminarily characterized. Each sample was aliquoted across six different concentrations and an *in vitro* egg hatching test (EHT) was conducted to evaluate the ovicidal effect on sheep GINs.

**Results:**

The results indicated that *Cichorium intybus* L. and *Foeniculum vulgare* Mill. extracts greatly inhibited egg hatching within 48 hours of exposure, showing efficacy (≥ 62.6%) at the three higher concentrations when compared with the other plants.

**Conclusion:**

The use of extracts of wild and cultivated perennial plant species in pastures can be a valid alternative to the use of synthetic anthelmintic drugs, which can generate problems, such as anthelmintic resistance, in the long term. Looking forward, further *in vitro* studies that evaluate the *in vitro* effect of these extracts on ruminant cell cultures, and field application through *in vivo* studies, would likely confirm the results obtained from preliminary *in vitro* EHTs. All these studies should be aimed at evaluating the therapeutic potential and future applicability of specific plant cultures in pastures to achieve sustainable pest control.

## 1 Introduction

Ruminant populations in rural areas are important resources for the economy of these regions, which often have complex developmental and socioeconomic problems. In zootechnical practices the quality and quantity of livestock-related production are conditioned by several biotic and abiotic factors, among which parasite infections are of great importance owing to their impact and wide diffusion to the extent that a pasture farm without parasites does not exist (Healey et al., 2018). Grazing is the main source of parasitic infestation in animals, so it is advisable to limit the parasite load in pastures. However, an infestation rate of zero is not pursued. The integrated approach to parasite management involves maintaining the natural host–parasite balance and rejects the vision of a complete elimination of parasites in animals. Complete elimination is impossible in grazing, and it has also been shown that a low parasite load in the animal helps to limit the occurrence of heavy infections (Healey et al., 2018; Kaplan, 2020).

The most common approaches to fixing these issues involve the use of drugs aimed at resolving the parasitosis symptoms, but without any preventive approach. An alternative to the use of medicines in livestock production should be sought in ethnobotanical traditions deeply rooted in rural areas, from which it is possible to derive answers after analyses are carried out with a scientific and modern approach. The tradition of collecting and using wild plants for several purposes is still deeply rooted in many rural communities, especially in less industrialized regions in which people have always been involved in primary sector production concerning crop production and livestock farming. This knowledge is often preserved in the oral tradition of rural communities, whose experiences, which are not fully known and supported by the scientific literature, constitute an unexplored heritage, especially in terms of bioactive compounds that can be extracted from wild plant resources.

Phytotherapy and ethnoveterinary medicine could solve or mitigate herd problems related to parasitosis by acting preventively with a wide selection of medicinal plants that combine versatile antimicrobial properties. A strong antioxidant capacity, the ability to positively modulate the micropopulation of the digestive tract, and increased immune defenses are only some examples of the benefits that could result from the careful use of selected plant species as a base or supplement in livestock feeding. All this translates into an improvement in the health status of the animals and their production performance (Bodas et al., 2012).

Focusing on sheep and goat farms, it is well known that these animals are widely affected by nematode-related parasitic problems, the most common of which can cause diseases in the stomach, intestine, liver, trachea, lungs, muscles, or skin of domestic ruminants (Perry and Randolph, 1999). Helminths, belonging to the group known as gastrointestinal nematodes (GINs), are the most widespread of all the parasites. They are present in almost all ruminant farms and cause the greatest economic losses, with a large impact on gross saleable product (Zanzani et al., 2014; Vande Velde et al., 2018; Rinaldi et al., 2022). GIN-related diseases are often the cause of slow and chronic stress that affects all animals on a farm, and often all farms in the area, making them more susceptible and less resistant to other diseases, primarily viral and bacterial infectious diseases. In daily practice, GIN control is managed through a series of pharmacological treatments carried out at various times of the year and without a precise diagnosis, thus using drugs inappropriately with the real risk of residues in meat, milk, and derivatives, as well as the massive dispersion of chemical elements into the environment. Avermectins can pollute drinking water and kill numerous species of invertebrates that are important for the balance of both aquatic and terrestrial ecosystems. Avermectins and pyrethrins (Rezende-Teixeira et al., 2022) are natural substances, yet their use has led to the development of resistance in pests, adverse effects in treated subjects, and ecotoxicity phenomena (Mukhopadhyay et al., 2022).

It is of great importance to study, experiment with, and test alternative strategies for GIN control, based on the use of bioactive phytoextracts from selected plant species with anthelmintic activity. It could also be important to determine the effectiveness of GIN infection prevention through direct livestock grazing of the botanical essences from which anthelmintic bioactive compounds can be extracted.

Local wild plants and their availability are of great interest, as they represent partially unexplored genetic resources both in terms of chemical composition and from an agronomic point of view in relation to their potential for greater adaptation to local environmental conditions. The characterization of these species is a fundamental step toward increased knowledge about these plants and their utilization on forage farms. Also, the use of plant extracts as phytotherapeutic products for livestock use not only represents a possible alternative to synthetic drugs and to the drug resistance of pathogens and parasites, but also supports consumer expectations of healthy food with high nutritional values that is produced in a sustainable way.

Pastures and meadow grasslands are often composed of annual and perennial plant species. The former complete their life cycle in one growing season and subsequently fruit and desiccate, whereas perennial species enter a dormant phase but remain available for grazing throughout the year. These perennial (or at least polyennial) species may represent a great resource of phytocomplexes that have not yet been widely explored, and their definition could lead to the identification of good practices to increase the general welfare of livestock through grazing. Studies carried out on various fodder species (both annual and perennial) have led to the individuation of tannins as the main molecular compounds responsible for this action (Schofield et al., 2001). Tannins are high-molecular-weight compounds. They are water-soluble polyphenols with the ability to precipitate proteins. They consist of large polyphenolic units and are generally divided into two broad categories: many plants contain those based on gallic acid and its metabolites and other plants contain those that are polyflavonoid in nature (so-called condensed tannins) (Tibe et al., 2011). The possibility of using them instead of synthetic compounds would allow their use as anthelmintics in the livestock sector, as these compounds have shown an important role in suppressing intestinal parasitosis in animals (Tibe et al., 2011). However, it is difficult to establish *a priori* whether or not the plant species can actually have a proven anthelmintic effect as a result of the presence of specific molecular classes. Laboratory tests that relate the effect of certain molecules (such as tannins) to the anthelmintic action they may have are related. However, anthelmintic action must often be translated into the effect of a single active ingredient rather than the effect of what is called a phytocomplex (Muñoz R de et al., 2021). Therefore, the use of plant species and their phytocomplexes must be combined with a chemical analysis that can characterize and direct research in translating the use of a specific plant species into anthelmintic action (Waller and Thamsborg, 2004; Githiori et al., 2006; Hoste et al., 2008).

The purpose of this study was to screen perennial plants that were surveyed and collected within the partner pastures after being cited in interviews conducted with ranchers and that were found to have an ethnoveterinary application in non-scientific and scientific reports. This screening was carried out with both biochemical and *in vitro* biological tests.

## 2 Materials and methods

**Figure 1:**
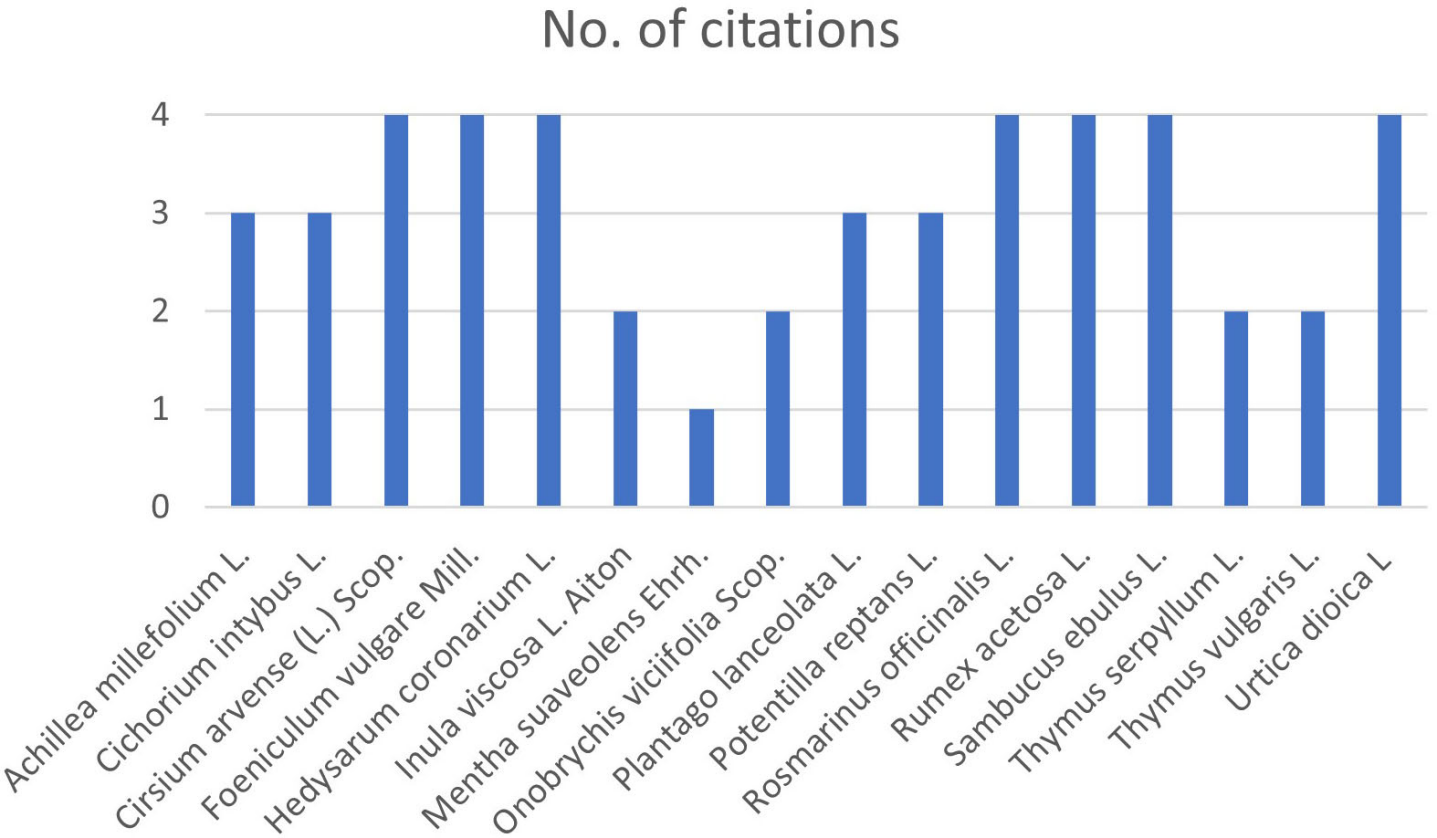
Graphical representation of answers on perennial plant species present in partner pastures.

In this study the possibility of using wild and cultivated perennial plant species as anthelmintic agents was explored, both through grazing and the extracted phytocomplex. The project partner farmers were interviewed using a unique framework (**Supplementary Table 1**) through which the information needed to identify the plant species that became the subject of this study was collected. The farmers’ answers were used to ascertain their knowledge of the native flora in the pastures of their herds and, following (i) head collection and identification with a dichotomous key (Pignatti et al., 2017) and (ii) attribution of the scientific name to the common or local name of the species (Acta Plantarum Staff, 2007), sixteen perennial or polyennial plant species were identified (**Figure 1**).

Sixteen different plant species were examined: *Achillea millefolium* L., *Cichorium intybus* L., *Cirsium arvense* (L.) Scop., *Foeniculum vulgare* Mill., *Hedysarum coronarium* L., *Inula viscosa* L. Aiton, *Mentha suaveolens* Ehrh., *Onobrychis viciifolia* Scop., *Plantago lanceolata* L., *Potentilla reptans* L., *Rosmarinus officinalis* L., *Rumex acetosa* L., *Sambucus ebulus* L., *Thymus serpyllum* L., *Thymus vulgaris* L., and *Urtica dioica* L. (**Table 1**). All samples were extracted via the conventional maceration technique and using three different solvents: distilled and deionized water (H_2_O), absolute ethanol (EtOH), and a hydroalcoholic ethanol: water mixture [EtOH:H_2_O, in a ratio of 8:2 v/v (volume to volume)]. Parameters such as pH, dry residue, and soluble solids content (°Bx) were quantified. The total phenolic content (TPC) was explored and quantified according to the Folin–Ciocȃlteu method (Dewanto et al., 2002). The obtained samples were aliquoted according to six concentrations (all aliquot concentrations are presented in **Supplementary Table 2**). In addition, an in vitro egg hatching test (EHT) (Coles et al., 1992) was performed in order to monitor the ovicidal action of the extracts of the studied plant species.

**Table 1:** List of sampled plant species.

Scientific name	Family	Order	Common name	Sampled parts	Origin
***Achillea millefolium* L.**	Asteraceae	Asterales	Yarrow	Leaves and roots	Pastures
***Cichorium intybus* L.**	Asteraceae	Asterales	Common chicory	Leaves and roots	Pastures
***Cirsium arvense* (L.) Scop.**	Asteraceae	Asterales	Field thistle	Leaves and roots	Pastures
***Foeniculum vulgare* Mill.**	Apiaceae	Apiales	Fennel	Leaves and roots	Pastures
***Hedysarum coronarium* L.**	Fabaceae	Fabales	Italia sainfoin	Leaves and flowers	CREA-ZA
***Inula viscosa* L. Aiton**	Asteraceae	Asterales	Enula bacicci	Leaves and roots	Pastures
***Mentha suaveolens* Ehrh.**	Lamiaceae	Lamiales	Round-leaf mint	Leaves	CREA-ZA
***Onobrychis viciifolia* Scop.**	Fabaceae	Fabales	Common sainfoin	Leaves and flowers	CREA-ZA
***Plantago lanceolata* L.**	Plantaginaceae	Lamiales	Ribwort Plantain	Leaves	CREA-ZA
***Potentilla reptans* L.**	Rosaceae	Rosales	Common cinquefoil	Leaves	CREA-ZA
***Rosmarinus officinalis* L.**	Lamiaceae	Lamiales	Rosemary	Leaves	CREA-ZA
***Rumex acetosa* L.**	Polygonaceae	Caryophyllales	Sorrel	Leaves	CREA-ZA
***Sambucus ebulus* L.**	Adoxaceae	Dipsacales	Elderberry	Leaves and roots	Pastures
***Thymus serpyllum* L.**	Lamiaceae	Lamiales	Wild thyme	Leaves	CREA-ZA
***Thymus vulgaris* L.**	Lamiaceae	Lamiales	Thyme major	Roots	CREA-ZA
***Urtica dioica* L.**	Urticaceae	Rosales	Nettle	Leaves and roots	Pastures

### 2.1 Chemicals and reagents

Ethanol ≥ 99.9% American Chemical Society (ACS) grade for the analysis was obtained from VWR International (Milan, Italy). Hydrochloric acid 37% Analytical Grade Reagents (RPE) for the analysis, anhydrous sodium carbonate (Na_2_CO_3_) for the analysis, gallic acid (C_7_H_6_O_5_) ACS grade for the analysis, methanol (CH_3_OH) for high performance liquid chromatography (HPLC), ethyl acetate (C_4_H_8_O_2_) ACS grade, 95% anhydrous *n*-hexane (*n*-C_6_H_14_), chloroform (CHCl_3_) for chromatography, and 1-butanol reagent (C_4_H_10_O) ACS grade ≥ 99.5% were purchased from Sigma-Aldrich Chemical Company (Milan, Italy). Folin–Ciocȃlteu reagent and acetonitrile hypergrade (C_2_H_3_N) for liquid chromatography–mass spectrometry (LC–MS) were purchased from Merck Millipore GmbH (Milan, Italy). 2,2-diphenyl-1-picrylhydrazyl (DPPH^·^) was obtained from Alfa Aesar (Thermo Fisher Scientific companies in Rodano, Milan, Italy).

Double-distilled water was used to prepare the solutions. The extracts obtained from the different plant species sampled were used without any purification and all the solutions analyzed, where necessary, were prepared by diluting the stock solutions of the extracts in water or a hydroalcoholic solution.

### 2.2 Plant materials: recovery and storage

The perennial plant species samples were collected after sampling was performed from June to September 2021 in the pastures and meadow grasslands of four selected livestock farms in the Campania region (in southern Italy) that mainly raise sheep and goats; the coordinates and locations of sampling areas are given below:

i) Azienda Agricola Mercorella Raffaele—San Giorgio la Molara (BN) (41°18′26.0″N 14°57′53.3″E, 840–860 meters above sea level (m a.s.l.);
ii) Azienda Agricola Di Santo Filomena—Guardia Lombardi (AV) (40°58′19.2″N 15°11′24.8″E, 785–905 m a.s.l.);
iii) Azienda Agricola De Leonardis Vito—Montecorvino Pugliano (SA) (40°38′38.6″N 14°56′32.2″E, 85 m a.s.l.); and
iv) Società Cooperativa Falode—Castello del Matese (CE) (41°24′54.8″N 14°25′14.1″E and 41°24′55.8″N 14°25′07.9″E, 1,005 and 1,030 m a.s.l., respectively).

Other selected perennial forage species were grown at the experimental station of CREA-ZA (Council for Research in Agriculture and Analysis of Agricultural Economics—Animal Husbandry and Aquaculture), S.S. 7 *Via* Appia, 85051 Bella Muro (PZ) (40°42′04″N 15°32′49″E).

The sampling areas were characterized by non-uniform climatic conditions ranging from temperate climate (iii) to humid (i, ii, and iv) conditions.

Sampling was carried out during the vegetation-flowering period (according to different plant species). At least 200 g per species was taken in order to collect enough sample to perform each type of analysis in triplicate. Both epigean and hypogean tissues were collected in all the samples, then mixed (leaves, stem, flowers if present, and roots were sampled). Plant samples were vacuum sealed directly into clean polyethylene bags and stored in refrigerated boxes at –4°C; they were brought to the laboratory within a maximum of 3 hours after collection.

### 2.3 Preparation of extracts

The plant samples were thoroughly washed with distilled water to remove impurities (dust, soil, and small insects). Each sample was dried in a ventilated oven at a temperature of 45°C for 48 hours and then minced using a scalpel and recovered with the use of a glass spatula. A triplicate extraction was performed for each sample. The samples were extracted using the conventional maceration technique; in addition, two different solvents and a combination of both were used: ethanol (EtOH) (henceforth referred to as E1), double-distilled water (H_2_O) (henceforth referred to as H1), and a solution of EtOH and H_2_O in an 8:2 (v:v) ratio (henceforth referred to as E8). Extractions were conducted at 25.00 ± 1.00°C at room temperature for 24 hours.

#### 2.3.1 Maceration extraction

The extraction time was chosen according the literature (Gaspar et al., 1997; Isbilir et al., 2012) and the group’s experience with extractions, with the aim of exhausting the plant matrix undergoing extraction in terms of bioactive compounds, but without the oxidization of the bioactive compounds of interest (polyphenols and molecules with anti-radical activity) by external agents. A total of 10 g of dried plant material was extracted by maceration with 100 mL of H_2_O, 100 mL of EtOH, and 100 mL of a solution of EtOH and H_2_O in a ratio of 8:2 (v:v) under continuous stirring and in the dark for 24 hours. After maceration, the extracts were filtered through paper to remove any floating substances. After filtration, the obtained extract in clear solution was brought to dryness with a rotary evaporator and subsequently stored in a dark container at –20°C.

### 2.4 Apparatus

A rotary evaporator HEIDOLPH Heizband Hei-VAP was used to perform all sample desolvations, equipped with a 2-L condensation chamber.

All absorbance measurements were performed by a MERCK Spectroquant^®^ Pharo 300 UV/Vis spectrophotometer. A 1.0-cm-long optical path glass cell was employed in all measurements.

The pH and temperature were determined by a de CRISON GLP 21 pH meter, a two-channel laboratory instrument.

A Brix and gravity refractometer with automatic temperature compensation (ATC) (with a detection range of 0%–32% for Brix grade and 1.000–1.130 for specific gravity) was used for specific gravity detection.

### 2.5 Soluble solid contents and pH

Soluble solid content (°Bx) and pH were measured in all extracts by means of a refractometer and CRISON pH meter.

### 2.6 Total phenolic compound content

The TPC was measured according to the Folin–Ciocȃlteu reagent method (Dewanto et al., 2002). For each sample extracted and subsequently dried, an aliquot of 1.0 mg (± 0.1 mg), weighed on an analytical balance, was taken and solubilized in a H_2_O:EtOH solution (with 5% EtOH). Both the weight of the initial sampling of extracted dried material (the same for all samples) and the dilution effect for analysis were taken into account in calculating the TPC value. Subsequently, 50 μL of resolubilized extract was added to a cuvette. A quantity of 2,300 μL of double-distilled water and 50 μL of Folin–Ciocȃlteu reagent were added and after 6 minutes 100 μL of sodium carbonate (Na_2_CO_3_) was added to the cuvettes. Each cuvette was shaken manually and allowed to stand for 90 minutes at room temperature. As the addition of sodium carbonate produces turbidity, which increases the absorbance signal, filtration of the solution was performed prior to absorbance measurement. Then, absorbance at 760 nm was measured and the total phenolic compound content was expressed as gallic acid-equivalent (GAE) concentration expressed in mol·L^-1^ using the calibration curve of gallic acid standard solutions (50–250 mg·L^–1^). All the measurements were made in triplicate and calculated as a mean value ± SD (*n* = 3).

### 2.7 Parasitological studies

#### 2.7.1 The *in vitro* egg hatch test

The main advantages of using *in vitro* tests to test the anthelmintic properties of plant species extracts are the low costs and rapid turnover, which allow for large-scale screening of plant compounds. The *in vitro* EHT is widely used in veterinary parasitology to test for anthelmintic (or “benzimidazole”) resistance and is also used to test potential new anthelmintic agents. This study evaluated the effect of selected plants when added to the diet of infected small ruminants. As will be detailed below, *in vitro* tests are performed using nematodes’ eggs extracted from fecal samples of infected animals and provide initial screening to establish concentrations of biologically active compounds before conducting *in vivo* animal tests, preserving the animal’s welfare as far as possible. *In vitro* anthelmintic activity is mainly validated through studies using various parasite models. One of these tests is the EHT, which is based on the capacity to inhibit the hatching of the parasite’s eggs (Coles et al., 1992). An EHT, modified according to the Regional Center for Monitoring of Parasitosis (CREMOPAR), in Eboli (Salerno Province, Campania Region, Italy) was used to determine the egg hatching inhibition efficacy of the selected plant extracts. The test is based on the practice of collecting GIN eggs directly from fecal samples collected rectally from naturally infected sheep. According to the protocol developed by Bosco et al. (2020) (Bosco et al., 2020), fecal samples were collected rectally from naturally infected sheep, stored at 10–15°C, and further processed as described below within 2 hours of collection. Fecal samples were homogenized and sieved using sieves with mesh sizes of 250, 125, 63, and 25 µm in descending order to separate GIN eggs from feces. The eggs were collected in the last sieve (25 µm). GIN eggs retained on the last sieve were washed and centrifuged for 3 minutes at 1,500 relative centrifugal force with distilled water, after which the supernatant was discarded. Following that, centrifugation was performed using a 40% sugar solution to float the eggs, which were then isolated in new tubes and mixed with distilled water. Egg solutions were centrifuged two more times to remove pellets and to obtain an aqueous solution with eggs. The separated and collected GIN eggs were used for *in vitro* tests. The tests were performed using 24-well plates. Approximately 200 eggs per well in 10 μL were incubated for 24 hours at 26°C in serial dilutions of 0.5% dimethyl sulfoxide (DMSO) (1 mL) of the studied plant species. The final concentrations of each plant extract used are reported in **Supplementary Table 2**. The concentrations that were used were the result of the maximum resolubilization (highest concentration per smallest volume used) specific to each plant species (and thus each extract) subjected to extraction. For each extract sample, six aliquots were obtained where concentration 6 was the most concentrated and concentration 1 was the least concentrated: specifically, the fold changes between solutions 1–6 are as follows: 1:32, 1:16, 1:8, 1:4, 1:2, and 1:1, respectively. Thiabendazole (0.025 mg·mL^–1^) and deionized water (0.5% DMSO) were used as positive and negative controls, respectively. After incubation for 48 hours at 27°C, the number of eggs and first-stage larvae (L1) were counted under an inverted microscope. The results were expressed as the mean percentage of eggs that hatched. Each concentration of each extract and the controls were analyzed in three replicates.

#### 2.7.2 Coprocultures

Given the differences in pathogenicity and response to anthelmintic molecules in GINs, the specific diagnosis of GINs is of significant importance. At present, the most widely applied method for this entails culture and microscopic analysis of third-stage larvae (L3), allowing for identification to at least the genus level.

During the study, in order to identify the GIN genera, larval cultures were performed, using a part of the fecal samples used for egg extraction in the *in vitro* EHT.

These samples were cultured following the protocol described by the Ministry of Agriculture, Fisheries, and Food (Friedhoff, 1978). Developed third-stage larvae (L3) were identified using the morphological keys proposed by van Wyk and Mayhew (2013) (Van Wyk and Mayhew, 2013). The genera that have been researched are those belonging to the strongyle group, such as *Trichostrongylus*, *Teladorsagia*, *Haemonchus*, *Cooperia*, *Oesophagostomum*, *Chabertia*, *Bunostomum*, and *Nematodirus*. Identification of each nematode genera was conducted on 100 L3 per sample; if a sample had 100 or less L3 present, all larvae were identified. Thus, based on the total number of larvae identified, it was possible to give the prevalence of each genus as a percentage.

### 2.8 Data analysis

The percentage values for egg hatching inhibition were calculated using a formula proposed by Coles et al. (1992) (Coles et al., 1992):


(1) $$\text{EHT }=\;\left[\frac{\left(\text{Number of eggs}\right)}{\left(\text{Number of larvae }+\text{ number of eggs}\right)}\right]\;\times \;100$$

For the comparison of values obtained for different concentrations and the controls within each extract, one-way analysis of variance (ANOVA) followed by Tukey’s test (*p*< 0.05) was performed.

## 3 Results

Information on ethnobotanical and ethnoveterinary applications of the selected and sparsely used plant species is mainly found in popular or traditional literature instead of in scientific literature, both in terms of uses and results. To this end, this work represents an initial screening from the perspective of TPC and EHT for the perennial species investigated. In general, the results obtained are an initial starting point to direct research into the use of extracts of wild and pasture-grown species as an alternative to the use of synthetic and non-synthetic veterinary drugs, generating greater heterogeneity of use and a possible reduction in drug resistance.

### 3.1 Farmer interviews

Interviews with ranchers were carried out prior to sampling and included the questions shown in **Supplementary Table 1**. We represented the collected responses in a single diagram shown in **Figure 1**: it can thus be inferred what plant species are present and worthy of interest within the pastures.

### 3.2 Parameters of the extracts

The extracts obtained were characterized by their weight, quantified using the dry residue, to evaluate the extracting capacity of the solvent and select the extraction technique. In addition, the extracted matrix samples were evaluated at the end of extraction and their actual depletion was verified in terms of total extract per quantity of solvent used. The values are shown in **Table 2**.

**Table 2:** pH, specific gravity, dry residue, and extract value of the sample extracts.

	H_2_O				EtOH				EtOH:H_2_O (8:2)			
Sample	pH	Brix (°Bx)	Residue (g·L^–1^)	Extract (g·g^–1^ DW)	pH	Brix (°Bx)	Residue (g·L^–1^)	Extract (g·g^–1^ DW)	pH	Brix (°Bx)	Residue (g·L^–1^)	Extract (g·g^–1^ DW)
***A. millefolium***	6.61 ± 0.01	1.8 ± 0.01*	35.9 ± 0.1	0.0503 ± 0.0018	6.44 ± 0.02	1.2 ± 0.01	47.1 ± 0.2	0.1980 ± 0.0007*	6.48 ± 0.03	1.0 ± 0.02	11.4 ± 0.2	0.2560 ± 0.0051*
***C. intybus***	6.75 ± 0.03	1.1 ± 0.01	18.7 ± 0.7	0.0373 ± 0.0008	6.58 ± 0.03	0.6 ± 0.01	21.8 ± 0.4	0.0752 ± 0.0021	6.58 ± 0.02	0.9 ± 0.02	15.4 ± 0.6	0.1310 ± 0.0012
***C. arvense***	6.47 ± 0.02	1.0 ± 0.02	33.5 ± 0.1	0.0612 ± 0.0033	6.57 ± 0.01	0.8 ± 0.01	30.6 ± 0.1	0.0336 ± 0.0011	6.62 ± 0.01	0.7 ± 0.01	43.0 ± 0.2	0.2510 ± 0.0023*
***F. vulgare***	6.61 ± 0.01	0.9 ± 0.01	20.4 ± 0.2	0.0408 ± 0.0027	6.81 ± 0.02	0.9 ± 0.01	3.10 ± 0.03	0.0452 ± 0.0014	6.57 ± 0.02	1.1 ± 0.01	1.40 ± 0.10	0.2020 ± 0.0011*
***H. coronarium* (Polla)**	6.53 ± 0.03	1.2 ± 0.01	78.4 ± 0.2	0.1570 ± 0.0021	6.79 ± 0.02	0.6 ± 0.02	15.8 ± 0.3	0.0377 ± 0.0022	6.49 ± 0.01	0.8 ± 0.01	71.0 ± 0.1	0.1210 ± 0.0014
***H. coronarium* (L)**	6.59 ± 0.03	1.1 ± 0.01	131.8 ± 0.9	0.2640 ± 0.0019*	6.78 ± 0.02	0.8 ± 0.01	37.6 ± 0.1	0.0369 ± 0.0008	6.51 ± 0.02	1.8 ± 0.01	65.4 ± 0.3	0.1930 ± 0.0019
***I. viscosa***	6.81 ± 0.01	1.5 ± 0.01*	24.4 ± 0.6	0.0488 ± 0.0055	6.72 ± 0.02	0.9 ± 0.01	36.3 ± 0.2	0.0316 ± 0.0006	6.65 ± 0.01	0.9 ± 0.01	43.8 ± 0.1	0.1420 ± 0.0010
***M. suaveolens***	6.63 ± 0.01	0.6 ± 0.01	48.0 ± 0.4	0.2400 ± 0.0011*	6.85 ± 0.01	1.7 ± 0.02*	7.40 ± 0.15	0.0176 ± 0.0015	6.81 ± 0.02	1.2 ± 0.01	38.5 ± 0.1	0.1520 ± 0.0004
***O. viciifolia* (Polla)**	6.54 ± 0.02	0.7 ± 0.01	141.0 ± 0.2	0.2820 ± 0.0042*	6.74 ± 0.02	1.4 ± 0.01	16.8 ± 0.2	0.0104 ± 0.0002	6.55 ± 0.01	1.3 ± 0.01	125.4 ± 0.9	0.0411 ± 0.0004
***O. viciifolia* (COV)**	6.76 ± 0.01	0.7 ± 0.01	12.8 ± 0.2	0.2560 ± 0.0005*	6.76 ± 0.03	1.0 ± 0.01	2.30 ± 0.04	0.0856 ± 0.0029	6.76 ± 0.01	1.2 ± 0.02	10.1 ± 0.3	0.1700 ± 0.0003
***P. lanceolata***	6.67 ± 0.01	1.1 ± 0.01	67.5 ± 0.1	0.3380 ± 0.0015**	6.62 ± 0.01	1.2 ± 0.01	39.6 ± 0.1	0.0860 ± 0.0019	6.47 ± 0.01	0.7 ± 0.01	51.2 ± 0.2	0.0612 ± 0.0008
***P. reptans***	6.34 ± 0.01	1.0 ± 0.02	18.7 ± 0.1	0.0935 ± 0.0004	6.58 ± 0.01	0.6 ± 0.01	2.10 ± 0.08	0.0659 ± 0.0030	6.49 ± 0.02	1.1 ± 0.02	8.20 ± 0.09	0.0160 ± 0.0009
***R. officinalis***	6.59 ± 0.01	0.8 ± 0.01	9.10 ± 0.15	0.0454 ± 0.0004	6.69 ± 0.02	0.7 ± 0.01	20.5 ± 0.3	0.1020 ± 0.0005	6.57 ± 0.03	0.6 ± 0.01	17.8 ± 0.3	0.0890 ± 0.0007
***R. acetosa***	6.55 ± 0.03	0.7 ± 0.01	19.3 ± 0.2	0.0964 ± 0.0032	6.72 ± 0.03	0.8 ± 0.01	17.1 ± 0.1	0.0725 ± 0.0024	6.80 ± 0.01	0.9 ± 0.01	33.9 ± 0.7	0.0876 ± 0.0006
***S. ebulus***	6.66 ± 0.01	0.9 ± 0.01	4.00 ± 0.07	0.0080 ± 0.0004	6.78 ± 0.01	0.9 ± 0.01	36.1 ± 0.1	0.0310 ± 0.0006	6.68 ± 0.01	1.4 ± 0.01	48.2 ± 0.2	0.0346 ± 0.0031
***T. serpyllum***	6.49 ± 0.02	0.8 ± 0.01	24.4 ± 0.2	0.1220 ± 0.0017	6.54 ± 0.02	1.1 ± 0.01	3.50 ± 0.05	0.0060 ± 0.0018	6.76 ± 0.03	1.2 ± 0.02	30.4 ± 0.1	0.0028 ± 0.0002
***T. vulgaris***	6.72 ± 0.01	0.8 ± 0.02	32.8 ± 0.1	0.1640 ± 0.0026	6.73 ± 0.03	1.8 ± 0.02*	7.50 ± 0.11	0.0435 ± 0.0034	6.57 ± 0.01	0.8 ± 0.01	24.2 ± 0.3	0.0308 ± 0.0018
***U. dioica***	6.52 ± 0.03	0.5 ± 0.01	20.6 ± 0.3	0.0412 ± 0.0014	6.65± 0.02	1.3 ± 0.02	15.5 ± 0.1	0.0722 ± 0.0021	6.60 ± 0.02	1.0 ± 0.02	17.3 ± 0.4	0.0963 ± 0.0016

Values are presented as mean (triplicate) ± SD. Asterisks indicate significant differences for parameters between the three solvents at a p-value< 0.05.

### 3.3 Total phenolic content

**Figure 2:**
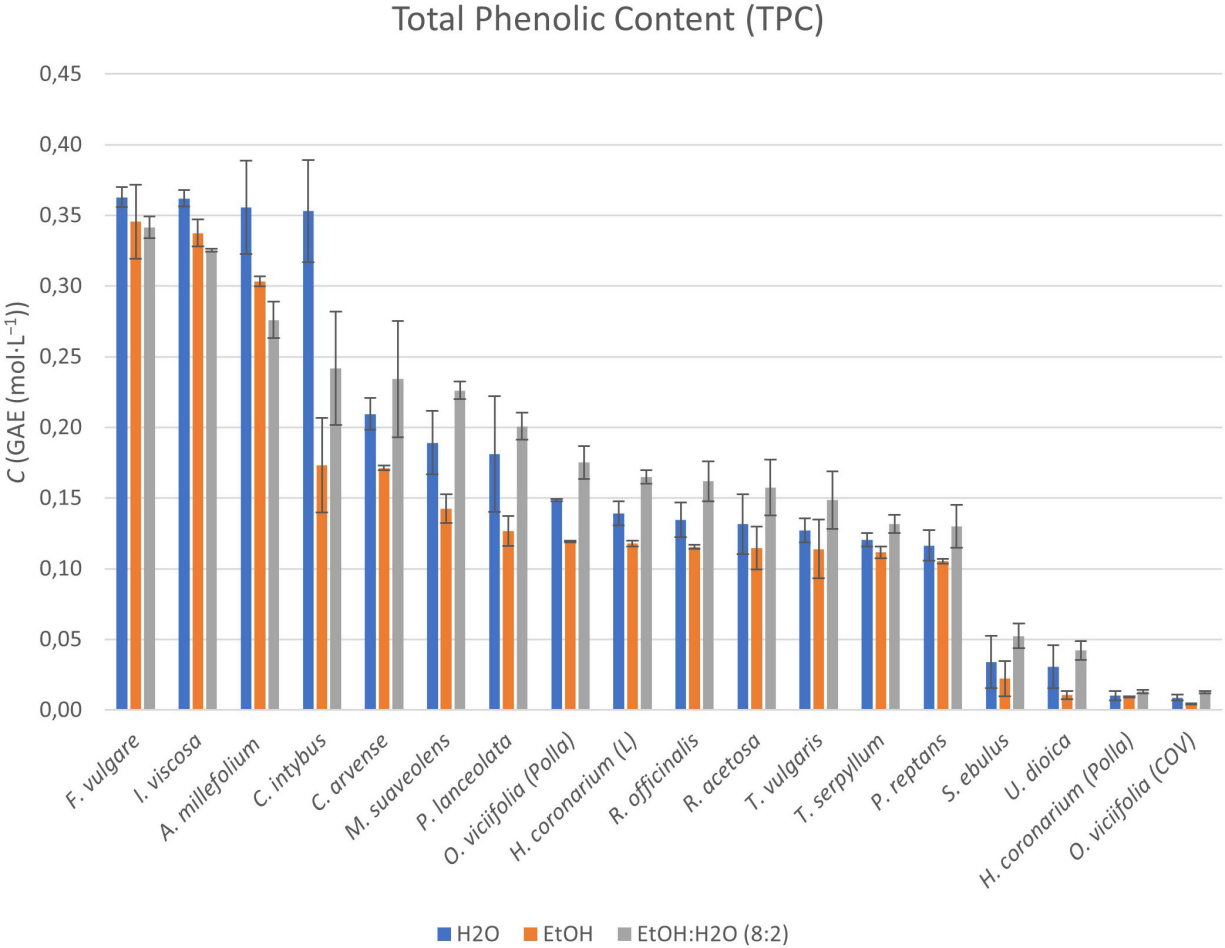
Graphical representation of TPCs within the extracts obtained from the selected and characterized perennial plants. Results are reported in concentration expressed in gallic acid equivalents (GAEs).

The total phenolic content (TPC) was analyzed in all samples and the results are shown in **Figure 2** (numerical data have been included in **Supplementary Table 3**).

The data obtained by comparing the three types of extracts for each plant matrix showed that there were important differences expressed in TPC, both with respect to the different solvents used and the different perennial plant species.

Of the 16 perennial plant species analyzed, *F. vulgare* and *I. viscosa* showed the highest TPC for all three solvents, with concentration values above 0.35–0.36 mol·L^-1^ (in GAE). Other species that showed similarly high concentration values for extracts in H_2_O were the species *A. millefolium* and *C. intybus*, with concentration values above 0.35 mol·L^-1^.

For extracts in EtOH, species such as *A. millefolium* exceeded TPC concentration values of more than 0.30 mol·L^-1^. Only the EtOH:H_2_O mixture extracts of the plant species *A. millefolium*, *C. arvense*, *C. intybus*, *M. suaveolens*, and *P. lanceolata* had TPC concentration values between 0.20 and 0.27 mol·L^-1^.

Extracts of the plant species *H. coronarium* (L), *M. suaveolens*, *O. viciifolia* (Polla), *P. lanceolata*, *P. reptans*, *R. acetosa*, *R. officinalis*, *T. serpyllum*, and *T. vulgaris* had TPC concentration values between 0.10 and 0.22 mol·L^-1^ in H_2_O, EtOH, and the EtOH:H_2_O mixture.

The extracts of the remaining plant species [*H. coronarium* (Polla), *O. viciifolia* (COV), *S. ebulus*, and *U. dioica*], on the other hand, all had TPC concentration values below 0.05 mol·L^-1^ for all solvents used.

### 3.4 Parasitological studies

#### 3.4.1 Egg hatching test

The EHT efficacy profile of each extract and each concentration, the positive control (TBZ), and the negative control (deionized water/DMSO 0.5%), as a percentage of eggs unhatched (mean of triplicates) is shown in **Table 3**. The results indicated that *C. intybus* and *F. vulgare* extracts greatly inhibited egg hatching within 48 hours of exposure, showing efficacy (≥ 62.6%) at the three higher concentrations when compared with the other plants. The main result of the EHT was that plant compounds with the highest TPCs also show the highest percentages of egg hatching inhibition.

**Table 3:** The percentages of egg-hatch inhibition of sheep gastrointestinal nematodes at different concentrations of tested extracts.

Plant	H_2_O						EtOH						EtOH:H_2_O (8:2)					
	Concentration						Concentration						Concentration					
	1	2	3	4	5	6	1	2	3	4	5	6	1	2	3	4	5	6
***A. millefolium***	0	0	0	61.5*	92.9*	98.8*	0	0	2.6	12.6	21.5	45.6	0	9.5	21.4	63.3*	90.5*	100.0*
***C. intybus***	6.6	12.6	43.4	70.7*	94.6*	97.3*	4.6	12.5	23.5	62.6*	64.3*	76.4*	3.6	8.2	13.6	87.5*	95.9*	98.0*
***C. arvense***	0	0	7.9	96.4*	99.3*	96.4*	0	0	6.4	7.1	14.5	47.1	0	0	0	58.6*	87.1*	92.1*
***F. vulgare***	12.6	34.6	85*	98.1*	98.8*	100.0*	11.8	42.6	43.7	67.5*	89.3*	97.5*	6.3	25.6	21.3	68.3*	71.9*	91.9*
***H. coronarium* (Polla)**	2.4	3.6	3.6	15.7	68.9*	72.7*	3.6	9.6	13.3	19.5	24.3	42.2	3.6	10.8	13.3	14.2	14.5	24.7
***H. coronarium* (L)**	0	40.0	15.2	72.4	89.7	93.8*	0	3.4	4.2	6.5	6.9	15.2	0	0	0	13.5	13.9	13.8
***I. viscosa***	0.4	1.6	4.7	4.1	5.3	64.1	1.6	7.8	12.1	62.3*	86.8*	95.4*	14.0	79.0	92.6*	96.3*	94.4*	98.1*
***M. suaveolens***	25.0	46.0	56.3*	77.6*	87.9*	98.3*	35.4	36.7	49.5	52.2*	59.5*	65.3*	40.2	43.6	45.8	49.4	49.8	55.6*
***O. viciifolia* (Polla)**	0	0.6	17.4	58.1*	94.2*	95.4*	0	0	0	6.7	8.9	23.6	0	9.7	12.8	12.9	11.6	21.3
***O. viciifolia* (COV)**	10.6	31.9	34.0	37.8	83.0*	87.2*	14.4	15.7	15.8	19.7	26.1	29.8	10.6	15.4	16.5	17.0	18.6	25.0
***P. lanceolata***	25.3	46.7	58.3*	68.5*	88.8*	98.9*	2.2	3.2	3.5	5.6	7.5	8.6	20.3	23.7	30.5	35	38.2	38.9
***P. reptans***	3.4	7.4	8.6	25.4	31.7	32.8	3.5	7.3	8.9	10.7	12.6	21.6	4.7	10.6	11.5	23.5	32.5	38.5
***R. officinalis***	18.8	28.5	30.5	35.4	38.8	46.8	16.7	18.7	20.0	25.4	30.7	36.7	30.3	40.7	40.0	47.0	47.7	51.0
***R. acetosa***	4.0	5.7	8.0	38.7	42.0	44.7	47.8	52.5	61.2	68.2	75.4	78.2	38.3	42.8	55.2	61.3	66.4	71.4
***S. ebulus***	2.2	3.8	6.5	7.1	7.6	8.7	4.3	30.4	37.0	39.1	45.7	53.8	8.7	34.2	52.2	60.3	78.3	87.5
***T. serpyllum***	3.4	3.9	4.2	7.6	10.7	15.8	5.2	7.3	7.8	11.8	15.4	25.7	5.7	6.8	10.7	11.6	12.8	18.7
***T. vulgaris***	2.1	3.5	4.6	5.6	15.7	21.6	3.2	5.8	7.5	8.3	10.8	12.4	4.6	7.4	10.5	11.6	15.7	24.7
***U. dioica***	9.6	15.4	29.8	42.6	82.4	86.7	0.5	1.0	1.2	3.9	7.8	15.6	0.2	0.6	1.2	2.4	4.7	9.4
**TBZ 0.025 mg·mL^–1^ (positive control)**	98.5*																	
**Deionized water (negative control)**	4.7																	

Asterisks indicate significant differences for parameters between the three solvents at a p-value< 0.05.

#### 3.4.2 Coprocultures

The genera of nematodes present were *Trichostrongylus* (33%), *Haemonchus* (31%), *Teladorsagia* (24%), and *Chabertia* (12%).

## 4 Discussion

Considering that one of the strategies to limit the use of synthetic anthelmintics in animal husbandry is the introduction of fodder containing condensed tannins into the diets of ruminants, it is well known that some perennial fodder legumes, such as *Hedysarum coronarium* L. and *Onobrychus viciifolia* L., are rich in these compounds, whose role in containing intestinal parasitosis is widely recognized (Manolaraki et al., 2010; Falcão and Araújo, 2011; Tibe et al., 2011). Exploring other plant species and referring to them in terms of TPCs has allowed us to group them together and evaluate the results obtained.

### 4.1 Extract parameters evaluation

Regarding the values monitored, the pH was in line with the extraction parameters, having in most cases a value close to neutrality for all samples obtained. Regarding the values related to specific gravity, expressed in Bx°, the values recorded were almost uniform, except in some cases: this means that the extracts obtained contain mostly compounds that do not belong to the carbohydrate family and therefore the amount of glycosylated molecules should be at a minimum (that is why the Bx° value is low), thus attributing the dry residue value calculated for the extract, a direct correspondence to the value obtained below for TPCs, as expressed in other works. (Saénz et al., 2009). This result, thus reported, helps us in the evaluation of the results related to TPC (see next section), and concerning the dry residue of the extracts and the relative amount of extract obtained per gram of plant matrix used in the extraction stage. A good result was obtained for the extracts of *P. lanceolata*, *O. viciifolia* (Polla e COV), *H. coronarium* (L), and *M. suaveolens*, with values by weight of 24%–34% of extract per matrix used in extracts in H_2_O. For extracts in EtOH, the value of extract weight was above 10% for *P. lanceolata* and *O. viciifolia* (Polla) only. In contrast, using the EtOH:H_2_O mixture (8:2), extracts with weight values between 12% and 26% were obtained for *P. lanceolata*, *O. viciifolia* (Polla and COV), *H. coronarium* (L and Polla), *M. suaveolens*, *T. vulgaris*, *T. serpyllum*, and *R. acetosa*.

### 4.2 TPC evaluation

In the evaluation of TPC, quantified by the Folin–Cioc̑ateu method and expressed in GAE (reported in mg∙L^-1^, as explicated and clarify in paragraph 2.6), interesting findings emerge from the data expressed in **Supplementary Table 3** and shown in **Figure 2**.

As shown, H_2_O appears in many cases to be the best solvent for the extraction of (poly)phenolic compounds from plant species such as *F. vulgare*, *I. viscosa*, *A. millefolium*, and *C. intybus*, especially in the case of the last one and with values above 0.35 mol·L^-1^ for all. For EtOH and EtOH:H_2_O (8:2) solvents, the extraction yield in terms of TPC was similar to and just below (0.34–0.27 mol·L^-1^) the values quantified for H_2_O in *F. vulgare*, *I. viscosa*, and *A. millefolium* species. For all other extracts of the studied plant species (still below 0.24 mol·L^-1^), the EtOH:H_2_O (8:2) mixture was the best solution among the extractive solvents investigated.

### 4.3 EHT evaluation

Anthelmintic resistance in ruminants is a severe and worsening problem worldwide and its development is a natural evolutionary process that is difficult to prevent if anthelmintics are overused/misused on the farm (Kaplan, 2020). This phenomenon and the risks associated with the presence of anthelmintic drug residues in the environment and animal food products have encouraged the search for alternative anthelmintic molecules (D’Ambola et al., 2018). Therefore, further research is needed to reduce the use of anthelmintics through the development of alternative approaches (Bosco et al., 2020). Several studies have shown the anthelmintic potential of plants for nematode control (Hoste et al., 2015; Castagna et al., 2019; Castagna et al., 2021; Ragusa et al., 2022) and, in particular, some *in vitro* studies highlight the inhibition of the hatching of GIN eggs caused by tannins contained in plants (Castagna et al., 2020).

In *in vitro* studies conducted in the same area (Castagna et al., 2020), hydroalcoholic extracts of *Isatis tinctoria* leaves and flowers were found to be highly effective in inhibiting GIN egg hatching in sheep. The authors consider the possibility of using the hydroalcoholic extracts for treating infected sheep or entire parts of *I. tinctoria* as a feed or dietary supplement in infected sheep for GIN control. In contrast, the study by D’Ambola et al. (2018) demonstrated weak ovicidal activity of *Hypoestes forskaolii* extracts against GIN eggs (D’Ambola et al., 2018).

Other studies have been conducted on the anthelmintic efficacy of *C. intybus*, but in large ruminants, that have also shown good efficacy against various nematode species (Peña-Espinoza et al., 2016; Peña-Espinoza et al., 2017; Peña-Espinoza et al., 2020). Some of the perennials tested in this study showed anthelmintic efficacy *in vitro*. In particular, the highest concentrations of all *C. intybus* and *F. vulgare* extracts demonstrated high (statistically significant) efficacy when compared with the control groups. Conversely, *A. millefolium*, *C. arvense*, *I. viscosa*, and *M. suaveolens* showed valid efficacy, with an egg hatch inhibition capacity of more than 50% in two extracts only. All other plant species, on the other hand, showed low efficacy.

## 5 Conclusions

The identification of potentially useful ethnoveterinary plant species is crucial for sustainable pasture management and current prevention guidelines. These species may not only provide useful phytoextracts against parasitic diseases but may themselves be species which, if eaten as forage, can prevent a wider range of diseases.

The research conducted has made it possible to link historical tradition, sometimes disseminated orally, to scientific literature. In fact, for many plant species analyzed based on information available to the farmers, a scientific explanation for their use was found based on bioactive content. The selected perennial plants present on the pastures of southern Italy could represent a resource for the control of helminths in ruminants. Indeed, it was possible to extract a satisfying amount of (poly)phenolic compounds from these plants by the maceration method and with three different solvents. For some plants, i.e., *F. vulgare*, *I. viscosa*, *A. millefolium*, and *C. intybus*, H_2_O was the best extraction solvent for these compounds. In particular, the significant *in vitro* ovicidal activity of extracts of *C. intybus* and *F. vulgare* shown in the present study highlights the anthelmintic potential of this alternative remedy to control GINs in sheep. However, further *in vivo* studies are needed to confirm the obtained results and evaluate therapeutic potential and future applicability.

## Data availability statement

The original contributions presented in the study are included in the article/**Supplementary Material**. Further inquiries can be directed to the corresponding author.

## Ethics statement

Ethics review and approval were not required for the animal study because ethics committee involvement was not necessary for this study. Written informed consent was obtained from the owners for the participation of their animals in this study.

## Author contributions

Conceptualization: PS, AB, GC, RS, and CG. Methodology: PS, AB, and RS. Validation: GC, GQ, SC, RS, and CG. Formal analysis: PS, AB, AP, AF, MM, and LR. Investigation: PS, AB, AP, AF, MM, and LR. Data curation: PS, AB, and RS. Writing—original draft preparation: PS, AB, and AP. Writing—review and editing: RS, CG, and GC. Supervision: SC, GQ, GC, RS, and CG. Project administration: SC, GQ, GC, RS, and CG. All authors contributed to the article and approved the submitted version.
