# Supplementary material for: Use of perennial plants in the fight against gastrointestinal nematodes of sheep
Source: Front Parasitol. 2023 Jun 2;2:1186149. doi: 10.3389/fpara.2023.1186149 (PMC11732003; doi:10.3389/fpara.2023.1186149)
Supplement: Supplementary file 1 [file Table_1.docx]

| Supplementary Table 1. List of questions asked during interviews with farmers. To all questions, the exclusive answer was between YES/NO: only question Q6 additionally included in consideration the identification of perennial plant species. | |
| --- | --- |
| ID | **Question** |
| Q1 | Does the farm have pastureland? |
| Q2.1 | (If the answer to Q1 is YES) Are the animals fed through grazing? |
| Q2.2 | (If the answer to Q1 is NO) Are the animals fed with packaged or baled feed? |
| Q3 | Does the farm have land for the fodder production used for animal feed on the farm? |
| Q4.1 | (If the answer to Q1 is YES) Is there spontaneous vegetation in the pastures? |
| Q4.2 | (If the answer to Q1 is YES) Are there plant species that are sown in the pastures only? |
| Q5 | (If the answer to Q4.1 is YES) Do the grazing animals like and eat the wild plant species found on the farm's pastures? |
| Q6 | (If the answer to Q4.1 is YES) Can you identify some or all of the wild species that animals eat when they go to pasture?  *If the answer to question Q6 is YES, the respondent is asked to indicate in the field or to indicate the common or vernacular name of the plant species referred to in question Q6* |
| Q7 | (If the answer to question Q6 is YES) Are these species present year-round and every year in the pasture? |
| Q8 | (If the answers to questions Q2.2 and Q6 are YES) Do you also use these species in the production of packaged or baled foods? |
| Q9 | Have you noticed whether there are variations (positive or negative) in the yields from the animals when they eat the identified wild species in pastures or used to produce packaged or baled food? |
| Q10 | Have you noticed any variations in the health (positive or negative) of the animals when they eat the identified wild species in pastures or used to produce packaged or baled food? |
