# Supplementary material for: Use of perennial plants in the fight against gastrointestinal nematodes of sheep
Source: Front Parasitol. 2023 Jun 2;2:1186149. doi: 10.3389/fpara.2023.1186149 (PMC11732003; doi:10.3389/fpara.2023.1186149)
Supplement: Supplementary file 2 [file Table_2.docx]

| **Supplementary Table 2.** The concentration of all extract obtained from perennial plant species selected and tested in the EHT assay. Concentrations were expressed in mg·mL^-1^. | | | | | | | | | | | | | | | | | | | | |
| --- | --- | --- | --- | --- | --- | --- | --- | --- | --- | --- | --- | --- | --- | --- | --- | --- | --- | --- | --- | --- |
| **Plant** | **H_2_O** | | | | | |  | **EtOH** | | | | | |  | **EtOH:H_2_O (8:2)** | | | | | |
|  | **Concentration** | | | | | |  | **Concentration** | | | | | |  | **Concentration** | | | | | |
|  | **1** | **2** | **3** | **4** | **5** | **6** |  | **1** | **2** | **3** | **4** | **5** | **6** |  | **1** | **2** | **3** | **4** | **5** | **6** |
| ***A. millefolium*** | 0.079 | 0.158 | 0.315 | 0.630 | 1.260 | 2.520 |  | 0.722 | 1.444 | 2.888 | 5.775 | 11.550 | 23.100 |  | 0.167 | 0.333 | 0.667 | 1.333 | 2.667 | 5.333 |
| ***C. intybus*** | 0.059 | 0.118 | 0.235 | 0.470 | 0.940 | 1.880 |  | 0.470 | 0.940 | 1.879 | 3.759 | 7.517 | 15.034 |  | 0.069 | 0.138 | 0.275 | 0.550 | 1.100 | 2.200 |
| ***C. arvense*** | 0.875 | 1.750 | 3.500 | 7.000 | 14.000 | 28.000 |  | 0.098 | 0.195 | 0.390 | 0.780 | 1.560 | 3.120 |  | 0.678 | 1.356 | 2.713 | 5.425 | 10.850 | 21.700 |
| ***F. vulgare*** | 0.064 | 0.128 | 0.255 | 0.510 | 1.020 | 2.040 |  | 0.063 | 0.125 | 0.250 | 0.500 | 1.000 | 2.000 |  | 0.025 | 0.050 | 0.100 | 0.200 | 0.400 | 0.800 |
| ***H. coronarium (Polla)*** | 1.563 | 3.125 | 6.250 | 12.50 | 25.00 | 50.00 |  | 0.001 | 0.004 | 0.016 | 0.064 | 0.256 | 1.024 |  | 0.001 | 0.006 | 0.036 | 0.216 | 1.269 | 7.776 |
| ***H. coronarium (L)*** | 1.563 | 3.125 | 6.250 | 12.50 | 25.00 | 50.00 |  | 0.001 | 0.004 | 0.016 | 0.064 | 0.256 | 1.024 |  | 0.001 | 0.006 | 0.036 | 0.216 | 1.269 | 7.776 |
| ***I. viscosa*** | 0.076 | 0.153 | 0.305 | 0.610 | 1.220 | 2.440 |  | 0.602 | 1.204 | 2.408 | 4.816 | 9.632 | 19.263 |  | 1.053 | 2.106 | 4.212 | 8.423 | 16.846 | 33.692 |
| ***M. suaveolens*** | 1.563 | 3.125 | 6.250 | 12.50 | 25.00 | 50.00 |  | 0.001 | 0.004 | 0.016 | 0.064 | 0.256 | 1.024 |  | 0.001 | 0.006 | 0.036 | 0.216 | 1.269 | 7.776 |
| ***O. viciifolia (Polla)*** | 1.563 | 3.125 | 6.250 | 12.50 | 25.00 | 50.00 |  | 0.001 | 0.004 | 0.016 | 0.064 | 0.256 | 1.024 |  | 0.001 | 0.006 | 0.036 | 0.216 | 1.269 | 7.776 |
| ***O. viciifolia (COV)*** | 1.563 | 3.125 | 6.250 | 12.50 | 25.00 | 50.00 |  | 0.001 | 0.004 | 0.016 | 0.064 | 0.256 | 1.024 |  | 0.001 | 0.006 | 0.036 | 0.216 | 1.269 | 7.776 |
| ***P. lanceolata*** | 1.563 | 3.125 | 6.250 | 12.50 | 25.00 | 50.00 |  | 0.001 | 0.004 | 0.016 | 0.064 | 0.256 | 1.024 |  | 0.001 | 0.006 | 0.036 | 0.216 | 1.269 | 7.776 |
| ***P. reptans*** | 1.563 | 3.125 | 6.250 | 12.50 | 25.00 | 50.00 |  | 0.001 | 0.004 | 0.016 | 0.064 | 0.256 | 1.024 |  | 0.001 | 0.006 | 0.036 | 0.216 | 1.269 | 7.776 |
| ***R. officinalis*** | 1.563 | 3.125 | 6.250 | 12.50 | 25.00 | 50.00 |  | 0.001 | 0.004 | 0.016 | 0.064 | 0.256 | 1.024 |  | 0.001 | 0.006 | 0.036 | 0.216 | 1.269 | 7.776 |
| ***R. acetosa*** | 1.563 | 3.125 | 6.250 | 12.50 | 25.00 | 50.00 |  | 0.001 | 0.004 | 0.016 | 0.064 | 0.256 | 1.024 |  | 0.001 | 0.006 | 0.036 | 0.216 | 1.269 | 7.776 |
| ***S. ebulus*** | 0.013 | 0.025 | 0.050 | 0.100 | 0.200 | 0.400 |  | 1.131 | 2.263 | 4.525 | 9.050 | 18.100 | 36.200 |  | 1.205 | 2.410 | 4.820 | 9.640 | 19.280 | 38.560 |
| ***T. serpyllum*** | 1.563 | 3.125 | 6.250 | 12.50 | 25.00 | 50.00 |  | 0.001 | 0.004 | 0.016 | 0.064 | 0.256 | 1.024 |  | 0.001 | 0.006 | 0.036 | 0.216 | 1.269 | 7.776 |
| ***T. vulgaris*** | 1.563 | 3.125 | 6.250 | 12.50 | 25.00 | 50.00 |  | 0.001 | 0.004 | 0.016 | 0.064 | 0.256 | 1.024 |  | 0.001 | 0.006 | 0.036 | 0.216 | 1.269 | 7.776 |
| ***U. dioica*** | 0.047 | 0.094 | 0.187 | 0.375 | 0.749 | 1.498 |  | 0.488 | 0.975 | 1.950 | 3.900 | 7.800 | 15.600 |  | 0.294 | 0.588 | 1.176 | 2.351 | 4.703 | 9.405 |
