# Supplementary material for: Use of perennial plants in the fight against gastrointestinal nematodes of sheep
Source: Front Parasitol. 2023 Jun 2;2:1186149. doi: 10.3389/fpara.2023.1186149 (PMC11732003; doi:10.3389/fpara.2023.1186149)
Supplement: Supplementary file 3 [file Table_3.docx]

| Supplementary Table 3. Total phenolic compound content (TPC) of the sample extracts. Values are presented as mean (triplicate) ± SD. Asterisks indicate significant differences for parameters between the three solvents at *p* < 0.05 | | | |
| --- | --- | --- | --- |
|  | **Total phenolic compound content (*C* GAE∙(mol·L^−1^))** | | |
| Sample | **H_2_O** | **EtOH** | **EtOH:H_2_O**  **(8:2)** |
| *A. millefolium* | 0.3558 ± 0.033** | 0.3033 ± 0.0037** | 0.2760 ± 0.013* |
| *C. intybus* | 0.3530 ± 0.036** | 0.1732 ± 0.0336 | 0.2419 ± 0.0402* |
| *C. arvense* | 0.20951 ± 0.01125* | 0.17132 ± 0.00163 | 0.2341 ± 0.0411* |
| *F. vulgare* | 0.3628 ± 0.0070** | 0.3455 ± 0.026** | 0.3415 ± 0.0078** |
| *H. coronarium* (Polla) | 0.13912 ± 0.08531 | 0.1179 ± 0.0021 | 0.16485 ± 0.04722 |
| *H. coronarium* (L) | 0.01015 ± 0.00324 | 0.00927 ± 0.00057 | 0.01318 ± 0.00123 |
| *I. viscosa* | 0.3620 ± 0.0058** | 0.3375 ± 0.0095** | 0.3253 ± 0.0009** |
| *M. suaveolens* | 0.1891 ± 0.0224 | 0.1423 ± 0.0102 | 0.22612 ± 0.0631* |
| *O. viciifolia* (Polla) | 0.00884 ± 0.00213 | 0.00424 ± 0.00037 | 0.01264 ± 0.00089 |
| *O. viciifolia* (COV) | 0.14862 ± 0.00085 | 0.11923 ± 0.00071 | 0.1751 ± 0.0115 |
| *P. lanceolata* | 0.18108 ± 0.04112 | 0.1267 ± 0.0107 | 0.2008 ± 0.0097* |
| *P. reptans* | 0.1164 ± 0.0109 | 0.1054 ± 0.0016 | 0.1300 ± 0.0153 |
| *R. officinalis* | 0.13442 ± 0.01228 | 0.1154 ± 0.0015 | 0.1618 ± 0.0142 |
| *R. acetosa* | 0.1315 ± 0.0213 | 0.1145 ± 0.0152 | 0.1574 ± 0.0198 |
| *S. ebulus* | 0.0340 ± 0.0185 | 0.02230 ± 0.0125 | 0.05243 ± 0.0087 |
| *T. serpyllum* | 0.1204 ± 0.0048 | 0.1116 ± 0.0042 | 0.1317 ± 0.0065 |
| *T. vulgaris* | 0.1271 ± 0.0087 | 0.11388 ± 0.0207 | 0.1485 ± 0.0204 |
| *U. dioica* | 0.03055 ± 0.001512 | 0.01060 ± 0.00287 | 0.04223 ± 0.00655 |
